# Beyond Neuronal Activity Markers: Select Immediate Early Genes in Striatal Neuron Subtypes Functionally Mediate Psychostimulant Addiction

**DOI:** 10.3389/fnbeh.2017.00112

**Published:** 2017-06-08

**Authors:** Ramesh Chandra, Mary Kay Lobo

**Affiliations:** Department of Anatomy and Neurobiology, University of Maryland School of MedicineBaltimore, MD, United States

**Keywords:** striatum, IEGs, psychostimulants, ∆FosB, c-Fos, Egr3, MSNs, cocaine

## Abstract

Immediate early genes (IEGs) were traditionally used as markers of neuronal activity in striatum in response to stimuli including drugs of abuse such as psychostimulants. Early studies using these neuronal activity markers led to important insights in striatal neuron subtype responsiveness to psychostimulants. Such studies have helped identify striatum as a critical brain center for motivational, reinforcement and habitual behaviors in psychostimulant addiction. While the use of IEGs as neuronal activity markers in response to psychostimulants and other stimuli persists today, the functional role and implications of these IEGs has often been neglected. Nonetheless, there is a subset of research that investigates the functional role of IEGs in molecular, cellular and behavioral alterations by psychostimulants through striatal medium spiny neuron (MSN) subtypes, the two projection neuron subtypes in striatum. This review article will address and highlight the studies that provide a functional mechanism by which IEGs mediate psychostimulant molecular, cellular and behavioral plasticity through MSN subtypes. Insight into the functional role of IEGs in striatal MSN subtypes could provide improved understanding into addiction and neuropsychiatric diseases affecting striatum, such as affective disorders and compulsive disorders characterized by dysfunctional motivation and habitual behavior.

## Introduction

Immediate early genes (IEGs) are activated transiently and rapidly throughout the brain by many cellular stimuli including psychostimulants. Traditionally, IEGs are used as markers of neuronal activity, in striatum and other brain regions, in response to psychostimulants. The striatum consists of the dorsal striatum, which regulates actions and habits vs. ventral striatum (a.k.a.- nucleus accumbens- NAc), which is involved in motivation and reinforcement (Voorn et al., 2004; Everitt and Robbins, 2013). Both striatal regions mediate psychostimulant-induced behavior as observed through motor, reward, motivational and habitual behaviors (Voorn et al., 2004). The main projection neurons in striatum are medium spiny neurons (MSNs), which are composed of two subtypes, those enriched in dopamine receptor 1 (D1) vs. dopamine receptor 2 (D2), as well as several other genes (Gerfen et al., 1990; Lobo et al., 2006; Heiman et al., 2008). The D1-MSNs vs. D2-MSNs are further distinguished by their projections through the brain (Gerfen, 1984, 1992; Smith et al., 2013). Early studies examining IEG gene and/or protein expression identified striatal MSN subtypes that are activated by psychostimulants (Robertson et al., 1991; Young et al., 1991; Berretta et al., 1992; Cenci et al., 1992; Moratalla et al., 1996; Bertran-Gonzalez et al., 2008). However, in focusing on IEGs as activity markers in MSN subtypes, important information about the functional role of IEGs in psychostimulant-mediated behavioral and cellular plasticity is potentially missed. This review article will discuss the subset of research addressing this issue by summarizing the current insight into IEG function in D1-MSN and D2-MSN subtypes in psychostimulant action. These findings have implications for addiction, as well as neuropsychiatric diseases affecting MSN subtypes including affective disorders and compulsive disorders.

## FosB in D1-MSNs as A Molecular Switch for Psychostimulant Addiction

The most well studied IEG in MSN subtypes is FosB. FosB is induced in striatum by acute cocaine (Hope et al., 1992) but the long lasting ∆FosB, generated from the FosB primary transcript (Yen et al., 1991), persistently accumulates after chronic psychostimulant exposure (Hope et al., 1994). The persistent accumulation of ∆FosB is a consequence of a lack of the degron domain containing C-terminal and through CAMKIIα phosphorylation at the Ser37 stabilization site in ∆FosB, thus producing this stable version of FosB (Carle et al., 2007; Robison et al., 2013). This long lasting induction of ∆FosB by cocaine is dependent on D1 receptor signaling (Moratalla et al., 1996) implicating this induction occurs primarily in D1-MSN subtypes. Recent studies using D1-GFP reporter lines confirm ∆FosB induction occurs primarily in D1-MSNs after chronic cocaine (Lee et al., 2006; Lobo et al., 2013). Consistent with these findings, FosB mRNA was induced in D1-MSNs with acute and chronic cocaine using a ribosomal tagging approach (Heiman et al., 2008; Chandra et al., 2015).

Initial studies using a tetracycline responsive promoter (TetOp)-∆FosB line crossed to a NSE-tetracycline transactivator (ttA) line resulted in expression of ∆FosB in striatal D1-MSNs (Kelz et al., 1999). This D1-MSN ∆FosB line displays enhanced locomotor and conditioned place preference (CPP) responses to cocaine (Table 1). Additionally, this line shows facilitated acquisition to cocaine self-administration at low threshold doses and enhanced effort to maintain self-administration of higher doses on a progressive ratio schedule of reinforcement (Colby et al., 2003; Table 1). These behaviors are occurring potentially through enhanced structural plasticity in D1-MSNs, since adenoassociated virus (AAV) mediated ∆FosB overexpression in NAc enhances MSN structural plasticity (Maze et al., 2010). Use of Cre-inducible herpes simplex virus (HSV) to overexpress ∆FosB in D1-MSNs in the NAc of D1-Cre mice confirmed the enhanced cocaine-mediated behavioral responses and showed that ∆FosB alone can enhance immature spine formation and reduce AMPAR/NMDAR ratios, in D1-MSNs (Grueter et al., 2013; Table 1). These structural and synaptic plasticity changes by ∆FosB are an indication of enhanced silent synapses, which are characteristic of cocaine effects on D1-MSNs (Graziane et al., 2016). Silent synapses are regarded as newly generated AMPAR-silent, NMDAR-only synapses, which are often present in the neonatal brain (Dong and Nestler, 2014). After withdrawal periods these synapses either retract or develop into fully functional synapses to induce new neural circuits (Dong and Nestler, 2014). A large body of evidence demonstrates that generation of these nascent synapses can promote behavioral responses to cocaine, such a locomotor sensitization, and that the maturation of these silent synapses can promote long-term behaviors associated with cocaine addiction, such as relapse (Russo et al., 2010; Dong and Nestler, 2014). Thus, ∆FosB may set the stage for long-term cocaine abuse by regulating the establishment of silent synapses in D1-MSNs during the initial stage of drug exposure. Whether ∆FosB in MSN subtypes continues to play a role in the long-term behaviors associated with drug addiction remains to be determined. Future studies performing these MSN subtype manipulations with prolonged abstinence and relapse models will help to answer this question. Finally, investigation of ∆FosB overexpression in D2-MSNs had no effect on cocaine-induced behaviors or spine formation but did enhance AMPAR/NMDAR ratios (Grueter et al., 2013; Table 1) suggesting that ∆FosB in these MSNs might play a role mature spine formation. This has implications for stress behavior since ∆FosB in increased in D2-MSNs in mice displaying stress susceptibility (Lobo et al., 2013).

**Table 1:** Medium spiny neuron (MSN) subtype manipulation of Immediate early genes (IEGs) in cocaine behaviors.

Molecule	Cell type	Brain region	Method	Effects mediated by cocaine	References
∆FosB	D1-MSN	Striatum	NSE-tTA × TetOp-∆FosB (Overexpression)	Increased CPP and locomotion	Kelz et al. (1999)
∆FosB	D1-MSN	Striatum	NSE-tTA × TetOp-∆FosB (Overexpression)	Enhanced cocaine acquisition and reinforcement (self-administration)	Colby et al. (2003)
∆FosB	D1-MSN	NAc	HSV-LS1-∆FosB + D1-Cre (Overexpression)	Increased locomotion and CPP	Grueter et al. (2013)
∆FosB	D2-MSN	NAc	HSV-LS1-∆FosB + D2-Cre (Overexpression)	No effect on locomotion and CPP	Grueter et al. (2013)
c-Fos	D1-MSN	Striatum	f/f-Fos-D1-Cre (Knockout)	Reduced locomotor sensitization, Reduced CPP extinction	Zhang et al. (2006)
Egr3	D1-MSN	NAc	AAV-Egr3-EYFP + D1-Cre (Overexpression)	Increased CPP and locomotion	Chandra et al. (2015)
Egr3	D2-MSN	NAc	AAV-Egr3-EYFP + D2-Cre (Overexpression)	Reduced CPP and locomotion	Chandra et al. (2015)
Egr3	D1-MSN	NAc	AAV-Egr3-microRNA + D1-Cre (Knockdown)	Reduced CPP and locomotion	Chandra et al. (2015)
Egr3	D2-MSN	NAc	AAV-Egr3-microRNA + D2-Cre (Knockdown)	Increased CPP and locomotion	Chandra et al. (2015)

**Figure 1:**
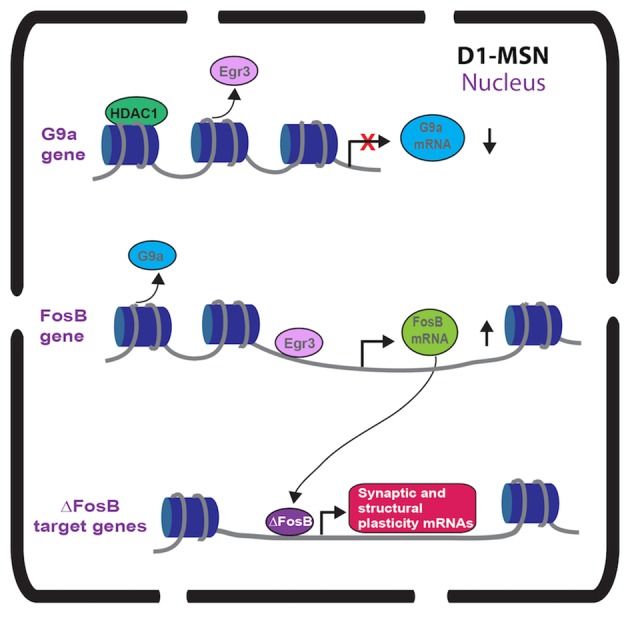
Immediate early gene (IEG) transcriptional regulation in nucleus accumbens (NAc) dopamine receptor 1 (D1)-medium spiny neurons (MSNs) after repeated cocaine. Repeated cocaine causes reduced Egr3 binding to the G9a promoter (Chandra et al., 2015) and G9a transcription is repressed by mechanisms including HDAC1 (Kennedy et al., 2013). This causes reduced G9a in NAc D1-MSNs (Chandra et al., 2015). Repeated cocaine results in increased Egr3 binding to the FosB promoter causing increased FosB in NAc D1-MSNs (Heiman et al., 2008; Chandra et al., 2015). The truncated FosB isoform, ∆FosB, is increased in NAc D1-MSNs (Lee et al., 2006; Lobo et al., 2013) after repeated cocaine leading to increased binding of ∆FosB on synaptic plasticity and structural plasticity gene promoters (Maze et al., 2010; Robison et al., 2013).

A mechanistic role of ∆FosB in promoting behavioral and structural plasticity after cocaine has been examined. The TetOp-∆FosB line displayed enhanced expression of GluR2 in NAc and GluR2 overexpression in NAc enhances cocaine CPP (Kelz et al., 1999). Robison et al. (2013) showed that ∆FosB increased CAMKIIα gene expression in NAc of the TetOp-∆FosB line and the enhanced cocaine-mediated behavioral and structural plasticity effects of ∆FosB in NAc are CAMKIIα dependent (Figure 1). Along with regulating CAMKIIα, ∆FosB regulates a number of genes in NAc by chronic cocaine (McClung and Nestler, 2003; Renthal et al., 2009). Investigation of ∆FosB in other brain regions demonstrated unique targets, such as CCK and Cdk5 (Chen et al., 2000; Vialou et al., 2014) suggesting that ∆FosB may differentially regulate transcripts in different cell subtypes. Thus, ∆FosB and other IEG transcription factors could differentially regulate gene transcription in D1-MSNs vs. D2-MSNs. Future studies using neuronal subtype ChIP can provide improved understanding into the MSN subtype transcriptional role of ∆FosB in cocaine action.

## c-Fos Function in D1-MSNs in Cocaine Action and c-Fos as An Activity Marker to Provide Insight into Function

While FosB has been the most widely studied IEG in striatal circuits in psychostimulant action, a functional role for c-Fos in D1-MSN subtypes has been investigated. Previous rat studies demonstrate c-Fos induction in both MSN subtypes when a psychostimulant is given in a novel environment (Badiani et al., 1998; Ferguson and Robinson, 2004). Using D1-GFP and D2-GFP reporter mice, researchers demonstrate c-Fos induction by cocaine in a novel environment, occurs primarily in D1-GFP MSNs throughout striatum with a small induction in D2-GFP MSNs in dorsal striatum (Bertran-Gonzalez et al., 2008). c-Fos deletion, in D1-MSNs, blunted cocaine-induced locomotor sensitization and MSN dendritic spine formation (Zhang et al., 2006; Table 1). Interestingly, c-Fos deletion in D1-neurons did not alter cocaine CPP but it did prevent the extinction of CPP. These data, illustrate a dynamic role for c-Fos induction in D1-MSNs, however, one cannot rule out the differential behavioral effects as being mediated by other brain regions that express the D1 receptor.

While, a focus on c-Fos as a neuronal activity marker is broadly utilized across neuroscience, researchers use this role of c-Fos to gain functional insight into striatal neuronal ensembles in psychostimulant exposure. c-Fos-lacZ or c-Fos-GFP rodents demonstrate active striatal neuronal ensembles in context dependent cocaine locomotor sensitization. Ablation of these neuronal ensembles in NAc prevents this context-dependent sensitization (Koya et al., 2009). While these c-Fos neuronal ensembles express both D1-MSN and D2-MSN markers, they express higher levels of a D1-MSN enriched gene, prodynorphin (Pdyn) and lower levels of D2-MSN enriched genes, D2 and adenosine 2A (A2A) receptor (Guez-Barber et al., 2011) suggesting a greater number of D1-MSNs in this population. The c-Fos activated NAc ensembles display silent synapses after cocaine locomotor sensitization, which is dependent on a context-specific sensitization (Koya et al., 2012; Whitaker et al., 2016). Future, studies using these c-Fos neuronal ensemble approaches that target MSN subtypes could delineate a functional role for D1-MSN vs. D2-MSN active neuron populations in psychostimulant action.

## A Bidirectional Role of Egr3 in Cocaine Action Through D1-MSN VS. D2-MSN Subtypes

While Egr1 (a.k.a. Zif-268) induction in striatum with acute psychostimulants is D1 receptor dependent (Daunais and McGinty, 1996; Steiner and Gerfen, 1996), there has been no investigation into a functional role of Egr1 in MSN subtypes. However, we recently examined the Egr family member, Egr3 in MSNs in cocaine action (Chandra et al., 2015). Egr3 is induced in total striatum with acute cocaine through the activation of D1 receptors (Yamagata et al., 1994; Jouvert et al., 2002). Using a ribosomal-trapping method we observed an induction of Egr3 mRNA in NAc D1-MSNs while a reduction occurred in D2-MSNS after repeated cocaine (Chandra et al., 2015). Mimicking the effects of cocaine, by enhancing Egr3 in D1-MSNs and reducing Egr3 in D2-MSNs in NAc using Cre-inducible AAVs combined with D1-Cre and D2-Cre lines, potentiated cocaine CPP and cocaine-induced locomotion. In contrast, blunting the effects of cocaine, reducing Egr3 in D1-MSNs and enhancing Egr3 in D2-MSNs, reduced these behaviors (Table 1). Egr3 binding is enriched on promoters of CAMKIIα and FosB in NAc and mRNA of these genes is enriched in NAc D1-MSNs after repeated cocaine (Chandra et al., 2015; Figure 1) suggesting that Egr3 acts as a potential upstream regulator of the ∆FosB and CAMKIIα mediated effects in D1-MSNs (Kelz et al., 1999; Grueter et al., 2013; Robison et al., 2013). Additionally, Egr3 binding is reduced on the promoter of the repressive histone methylation enzyme, G9a and G9a is reduced in NAc D1-MSNs after repeated cocaine (Chandra et al., 2015; Figure 1). G9a binding and its histone mark, H3K9me2, is reduced on the FosB promoter after repeated cocaine and/or in the Tet-Op-∆FosB D1-MSN line (Maze et al., 2010; Figure 1). Further, G9a overexpression prevents dendritic spine induction by repeated cocaine (Maze et al., 2010). Thus, Egr3 may act, in D1-MSNs, as an upstream regulator of ∆FosB induction and structural plasticity by direct transcriptional regulation at the FosB promoter and indirect regulation through reduced binding at the G9a promoter. This potentially leads to repression of G9A transcription through other factors, such as HDAC1 (Kennedy et al., 2013; Figure 1). Interestingly, a previous study showed reduced G9a in both D1-MSNs and D2-MSNs in the entire striatum after repeated cocaine and the effects of G9a were mediated through D2-MSNs (Maze et al., 2014). However, this could be a consequence of a G9A developmental knockout in MSN subtypes, which indeed led to D2-MSNs displaying a D1-MSN subtype identity. Finally, we recently demonstrated that Egr3 binding on the peroxisome proliferator-activated receptor-gamma coactivator (PGC)-1α promoter was increased after repeated cocaine exposure (Chandra et al., 2017). We observed an increase of PGC-1α in D1-MSNs and a reduction in D2-MSNs after repeated cocaine. Consistent with these findings, we observed bidirectional behavioral outcomes to cocaine when PGC-1α was overexpressed in NAc D1-MSNs vs. D2-MSNs. Although, the function of PGC-1α in MSN subtypes in cocaine action is unclear, previous research shows that PGC-1α can mediate dendritic spine plasticity in neurons (Cheng et al., 2012). Examination of Egr3’s role in MSN structural and synaptic plasticity, as well as the role of G9a, ∆FosB and PGC-1α in mediating these effects through Egr3 will be important for understanding the cellular role of Egr3 in cocaine action.

## Conclusion

While studies examining psychostimulant-mediated IEG function in D1-MSN vs. D2-MSN subtypes are sparse, they have provided some insight into mechanisms by which IEGs act in these neuron subtypes. This includes actions primarily through D1-MSNs in psychostimulant-mediated molecular, cellular and behavioral plasticity. Overall we focus on three select IEGs that have been examined in MSN subtypes in psychostimulant action. However, examination of other psychostimulant relevant IEGs, such as Arc and CREB (Carlezon et al., 1998; Salery et al., 2017), in MSN subtypes will provide a more comprehensive understanding of IEG function in striatal neuron subtypes in psychostimulant abuse. These MSN subtype specific studies have been restricted to non-contingent behaviors or the acquisition phase of self-administration, in the case of ∆FosB. Nonetheless, they provide potential mechanistic insight into the early stages of drug exposure before the shift to the addictive state. ∆FosB is involved in the generation of early plasticity processes, such as silent synapses, in D1-MSNs in psychostimulant exposure that set the stage for long-term neural circuit reorganization and the enduring behaviors occurring in addiction. These processes occur in c-Fos expressing neurons and Egr3 has been shown to transcriptionally regulate FosB, as well as molecules involved with structural plasticity. Thus c-Fos and Egr3, along with ∆FosB, likely play a role, in D1-MSNs, in mediating the nascent spine formation in the early stages of drug abuse that can ultimately give rise to stable spines and the long-term behaviors associated with addiction. Whether these IEGs are necessary to maintain the long-term circuit remodeling and relapse behaviors in addiction remains to be determined. Future studies examining these IEGs in MSN subtypes using more relevant models of addiction including self-administration with abstinence and relapse behavior will provide improved understanding of IEGs in striatal MSN subtypes in addiction. Finally, while studying these IEGs alone has provided important information, a more detailed probing of IEG transcriptional targets in MSN subtypes after drug exposure will provide improved information into the functional consequence of IEGs in addiction. Insight into how these IEG targets are regulating synaptic plasticity, structural plasticity, neural circuit remodeling and ultimately behavior could provide potential molecules that could be therapeutically targeted in addiction. These studies also have implications for neuropsychiatric diseases affecting striatal based behavior, including affective disorders and compulsive or stereotypy disorders.

## Author Contributions

MKL and RC both contributed to the writing of the manuscript, as well as the preparations of the figure and table.

## Conflict of Interest Statement

The authors declare that the research was conducted in the absence of any commercial or financial relationships that could be construed as a potential conflict of interest.
